# Biomechanical analyses of the handstand: a systematic review

**DOI:** 10.3389/fspor.2025.1694648

**Published:** 2025-12-15

**Authors:** Mahri McDonald, Julien S. Baker, Yaodong Gu, Ukadike C. Ugbolue

**Affiliations:** 1 Faculty of Sports Science, Ningbo University, Ningbo, China; 2 Division of Sport and Exercise, School of Health and Life Sciences, University of the West of Scotland, Glasgow, United Kingdom; 3 Centre for Health and Exercise Science Research, Department of Sport, Physical Education and Health, Hong Kong Baptist University, Kowloon Tong, Hong Kong SAR, China

**Keywords:** gymnastics, kinetics, kinematics, EMG, center of pressure, base of support

## Abstract

**Purpose:**

The aim of this systematic review was to investigate the biomechanical analysis of the handstand.

**Methods:**

Systematic searches were conducted using databases SPORTDiscus, ScienceDirect, and PubMed up to August 2025. The search included the following terms: “EMG or Electromyography and Handstand AND Gymnastics,” “Kinetics AND Kinematics and Handstand AND Gymnastics,” “Kinetics AND Handstand,” and “Kinematics AND Handstand.” Initially, 491 publications were identified. Of these, 21 studies were included in this systematic review, each of which conducted a biomechanical analysis of the handstand. Data from the 21 articles were extracted to conduct a biomechanical analysis of the handstand.

**Results:**

Of the included studies, 31% analyzed balance control strategies of the handstand, 57% performed a kinetic and kinematic analysis of the handstand, and 10% compared muscle activity during the handstand. Furthermore, 42% of the publications analyzed the center of pressure of the handstand, 36% analyzed joint angles, 21% reported on velocity, 21% examined joint torque contributions, 15% investigated angular velocity, 10% analyzed ground reaction forces (GRFs), 10% investigated electromyography (EMG) data, and 5% assessed vertical ground reaction forces (vGRFs).

**Conclusion:**

The findings of this study indicate that gymnasts minimize problems related to disturbed balance during the handstand using a “wrist strategy” to secure a fixed-body configuration. If the “wrist strategy” fails, then the wrists, shoulders, hips, and elbows are utilized, which can be interpreted as a mixed control strategy. Visual conditions and head positions also affect handstand balance. In particular, closing the eyes leads to decreased stability, and neck flexion impairs performance during the handstand. Gymnasts with greater strength possess better balance control. Other factors that also influence handstand performance are participation level and age of the performers.

## Introduction

A handstand, although considered to be a basic element in gymnastics, is one of the most important foundational skills. It provides a progression route for transferable skills on different apparatuses (i.e., rings, beam, uneven bars, and parallel bars) and across various floor disciplines (1). The handstand can be characterized as the action of maneuvering the body into a fixed inverted vertical position while balancing on the hands. However, this maneuver can be made significantly challenging when performed using specific apparatuses or under conditions that affect overall balance and mechanical characteristics of the movement (2). The challenging conditions of the handstand become apparent due to a reduced base of support and an increased distance between the base and the center of gravity as a result of supporting the body with extended arms, which decreases stability (3). In acrobatics and competitions, gymnasts performing on rings, parallel bars, and balance beams are evaluated by judges based on their overall technique, with the main focus on stability during a handstand; therefore, effective balancing strategies are crucial (4). Skills related to handstands are examined in this study because they offer a unique and demanding model for investigating human balance, motor control, and postural regulation under inverted conditions. Moreover, handstands require complex neuromechanical coordination, as they provide a controlled setting for studying variability, causality, and joint contributions to balance. Handstands help test theories of motor control, including concepts of degrees of freedom and adaptive variability. Aside from being part of a gymnastics act, handstands provide insight into the sophisticated systems that govern human movement and balance.

When balancing during the handstand, multiple muscular mechanical systems become activated, providing performers with signals and information from visual, proprioceptive, and vestibular cues (5). Visual systems have been identified as playing a fundamental role in handstand performance, as visual information provides essential temporal and spatial coordination data; this information provides immediate feedback and guides the control of movement and movement trajectory (6). The vestibular apparatus helps the body preserve its postural equilibrium and plays a crucial role in coordinating head positioning (7). However, when its function is compromised, balance is disrupted, often due to changes in head positioning, which can have a detrimental effect on handstand performance (4). Proprioception, defined as the ability of the body to distinguish and interpret its position in space, provides essential information about the orientation of different body parts. Proprioceptive awareness includes sensations of motion, tendon and muscle tension, pressure, and equilibrium, all of which originate from sensory neuronal pathways in the skin, muscles, and joint tissues (8). Proprioception plays a critical role in handstand performance, as it governs the velocity and type of muscle responses required for balance; however, the overall functioning of proprioception may vary depending on the training experiences of beginner and elite gymnasts (9).

Successful handstand performance and optimal balance are influenced by prominent joint actions, movement patterns, and the activation of specific muscle groups. This coordination is accompanied by wrist and finger flexion, which form part of the wrist strategy of a gymnast. Further joint actions include cervical extension, which involves a slight tilt of the head to allow the gymnast to view the anchor point between the hands; shoulder girdle elevation, in which the gymnast pushes through the shoulders to eliminate gaps between the shoulders, arms, and ears; extended hips with slight external rotation; posterior pelvic tilt, which helps flatten the lower back; knee extension, which supports the gymnast in maintaining straight legs; and planter flexion, culminating in pointed toes (10).

Moreover, researchers have conducted a thorough biomechanical analysis of the handstand. From a mechanical perspective, the handstand is usually regulated by the elevation of the center of gravity, the overall difficulty of maintaining balance, and the size of the base of support. Using force plates, 2D/3D motion analysis systems, and electromyography, it is possible to distinguish several biomechanical strategies involved in maintaining balance during a handstand and further determine the mechanical characteristics. Biomechanical analyses provide essential information about coordinated joint motions at the hips, shoulders, and wrists (11), as well as center of pressure (COP) shifts, which are recorded in the anterior–posterior (AP) and medial–lateral (ML) directions (3). The scientific literature has reported biomechanical analyses investigating COP movements alongside integrated studies of head position, visual conditions, and angular differences between body segments (5, 12, 13). Studies generally highlight the influence of the “wrist strategy,” which correlates with better balance control when the COP shifts toward the wrist joints. Balance control is maintained by maximizing balance using the wrist joints. This action results in increased pressure on wrist joints, causing the center of gravity to move toward the wrists. When performers are unable to effectively implement the “wrist strategy,” then either the “shoulder strategy” or the “hip strategy” may be used, although these alternatives often result in decreased balance and technical precision (6, 11, 14). Furthermore, research has also explored the physiological characteristics of participants performing handstands, with a focus on specific characteristics and a comparison of the techniques used during performance. These studies have examined strength parameters and their effects on handstand performance, compared control strategies employed by less experienced and highly experienced gymnasts, and analyzed differences in handstand performances between junior and adult gymnasts (2, 14–16).

Therefore, the aim of this systematic review was to conduct a biomechanical analysis of the handstand, investigating the mechanical factors influencing handstand performance among gymnasts. The secondary aim was to examine how biomechanical variables differed among participants.

## Methods

### Eligibility criteria

This systematic review was conducted following the PRISMA (Preferred Reporting Items for Systematic Reviews and Meta-Analyses) guidelines. The inclusion criteria were as follows: (1) studies incorporating EMG, (2) studies examining the kinematics or kinetics of handstand performance, (3) articles published in English, and (4) studies investigating gymnast performances across all levels, sexes, and age groups. The exclusion criteria were as follows: (1) articles focused on coaching handstands rather than investigating performance outcomes, (2) studies that did not investigate handstand performance, (3) studies that did not include kinematics, kinetics, or EMG, (4) studies that were in languages other than English, and (5) studies involving participants with no gymnastics experience. Studies were required to evaluate the kinetics, kinematics, or EMG of handstand performance [e.g., using two-dimensional (2D) video analysis systems, force platforms, three-dimensional (3D) motion analysis systems, or EMG assessment]. Furthermore, studies were required to report descriptive data (e.g., mean values, standard deviations, and sample sizes).

### Information sources

The databases SPORTDiscus, ScienceDirect, and PubMed were searched up to August 2025. The search terms utilized were “EMG or Electromyography and Handstand AND Gymnastics,” “Kinetics AND Kinematics and Handstand AND Gymnastics,” “Kinetics AND Handstand,” and “Kinematics AND Handstand.”

### Study selection

Once the database searches were completed, articles were imported into reference manager EndNote. All duplicate articles were removed. All titles and abstracts were screened for eligibility, and articles meeting the inclusion criteria were retrieved. The full-text articles were then assessed using the eligibility criteria. The retrieved studies were published between November 1988 and August 25, 2025.

### Data collection process

Data from the retrieved articles were extracted for biomechanical analysis of handstand performance. The extracted information was organized into a summary table presenting the relevant findings, study objectives, and analytical methods. The following details were extracted from each study: study aim, sport investigated, participant characteristics, sample size, demographics (age, gender, mass, and height), measurement systems used (e.g., 2D video analysis, 3D motion analysis, and surface or intramuscular EMG), and participant setup.

### Quality assessment

 Table 1 illustrates the methodological rigor of the studies assessed in this systematic review and provides an overview of the quality of all included publications (33). The McMaster Critical Appraisal Tool was utilized for all publications, and a quality score was assigned accordingly. The tool consists of 16 items, with each criterion receiving a score of “1” if an item is met and “0” if not met. The maximum possible score was 16, and scores were categorized as follows: excellent (15–16), very good (13–14), good (11–12), fair (9–10), and poor (≤8).

**Table 1:** Methodological rigor of the included studies using the McMaster critical review form for quantitative analysis.

Questions	(5)	(15)	(16)	(6)	(17)	(4)	(12)	(18)	(19)	(20)	(21)	(22)	(13)	(14)	(23)	(24)	(25)	(26)	(27)	(11)	(28)
1. Was the purpose stated clearly?	1	1	1	1	1	1	1	1	1	1	1	1	1	1	0	0	1	1	1	1	1
2. Was relevant background literature reviewed?	1	1	1	1	1	1	1	1	1	1	1	1	1	1	0	1	1	1	1	1	1
3. Was the study design stated?	1	1	1	1	1	1	1	1	1	1	1	0	0	1	0	0	0	0	1	0	1
4. Was the sample described in detail?	1	1	1	1	1	1	1	1	1	1	1	1	0	1	1	1	1	1	1	0	1
5. Was the sample size justified?	1	1	1	1	1	0	1	1	1	1	1	1	0	1	1	1	0	0	1	1	1
6. Were the outcome measures reliable?	1	1	1	1	1	1	0	1	1	1	1	1	1	1	0	0	1	1	1	1	1
7. Were the outcome measures valid?	1	1	0	1	1	1	1	1	1	1	1	1	1	1	0	1	1	1	1	1	1
8. Was the intervention described in detail?	0	0	0	0	0	1	0	1	1	1	0	1	0	0	0	1	0	0	0	0	1
9. Was contamination avoided?	0	0	0	1	0	0	0	0	0	1	0	1	0	0	0	0	0	0	1	0	1
10. Was co-intervention avoided?	1	1	1	1	1	1	1	1	1	1	1	0	1	1	1	1	0	0	1	1	1
11. Were the results reported in terms of statistical significance?	1	1	1	1	1	1	1	1	1	1	1	1	1	1	1	1	1	1	1	1	1
12. Were the analytical methods appropriate?	1	1	1	1	1	1	1	1	1	1	1	1	1	1	1	1	1	1	1	1	1
13. Was the clinical importance reported?	0	0	0	0	0	0	0	0	0	0	0	0	0	0	0	0	0	0	0	0	0
14. Were dropouts reported?	0	0	0	0	0	0	0	1	1	0	1	0	0	1	0	0	0	0	0	0	0
15. Were the conclusions appropriate, given study methods and results?	1	1	1	0	1	1	1	1	1	1	1	1	1	1	1	1	1	1	1	1	1
16. Were the study limitations acknowledged?	0	0	1	0	0	0	1	1	1	1	1	0	0	0	0	0	0	0	1	1	1
Result	11	11	11	11	11	10	11	14	14	14	13	12	8	12	6	9	8	8	14	10	14
Descriptor	Good	Good	Good	Good	Good	Good	Good	Very good	Very good	Very good	Very good	Good	Poor	Good	Poor	Poor	Poor	Poor	Very good	Good	Very good

## Results

### Literature search and retrieval

**Figure 1:**
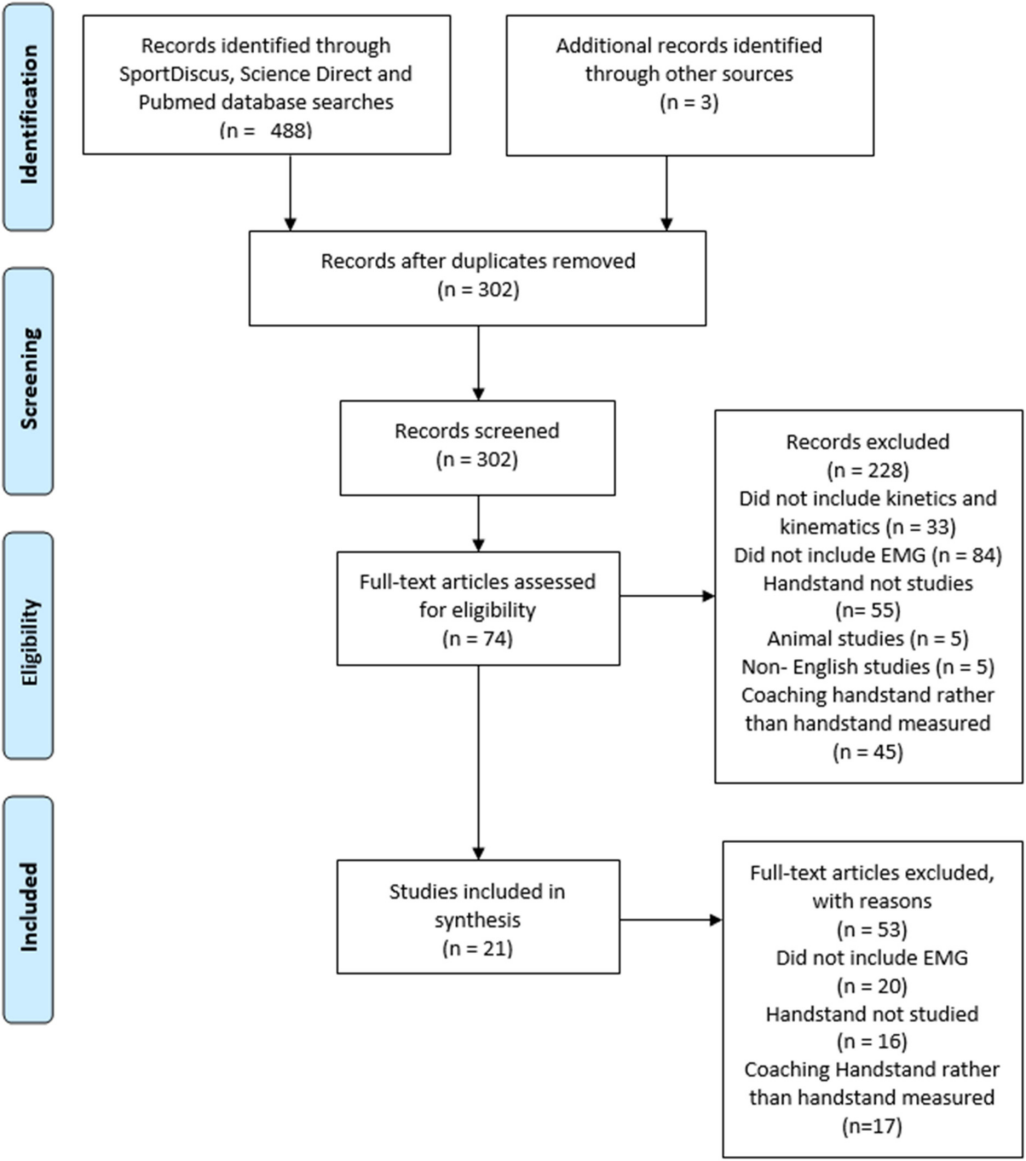
PRISMA flowchart.

Figure 1 presents the PRISMA flowchart outlining the search methodology. A total of 489 titles were initially retrieved. After removing duplicates using reference management software (EndNote X9), 302 papers remained for screening. A total of 228 records were eliminated. A keyword search for “animal” eliminated five studies. The remaining 223 papers were screened further, of which 33 were excluded for not including kinetics or kinematics, 84 for lacking EMG data, 56 for not investigating the handstand, 5 for being non-English, and 45 for focusing on handstand coaching rather than movement measurement. The remaining 74 studies underwent full-text review, after which 53 were removed for not meeting the inclusion criteria. Ultimately, 21 articles met the inclusion criteria and were included in this systematic review.

### What were the purposes and most frequent topics examined?

**Figure 2:**
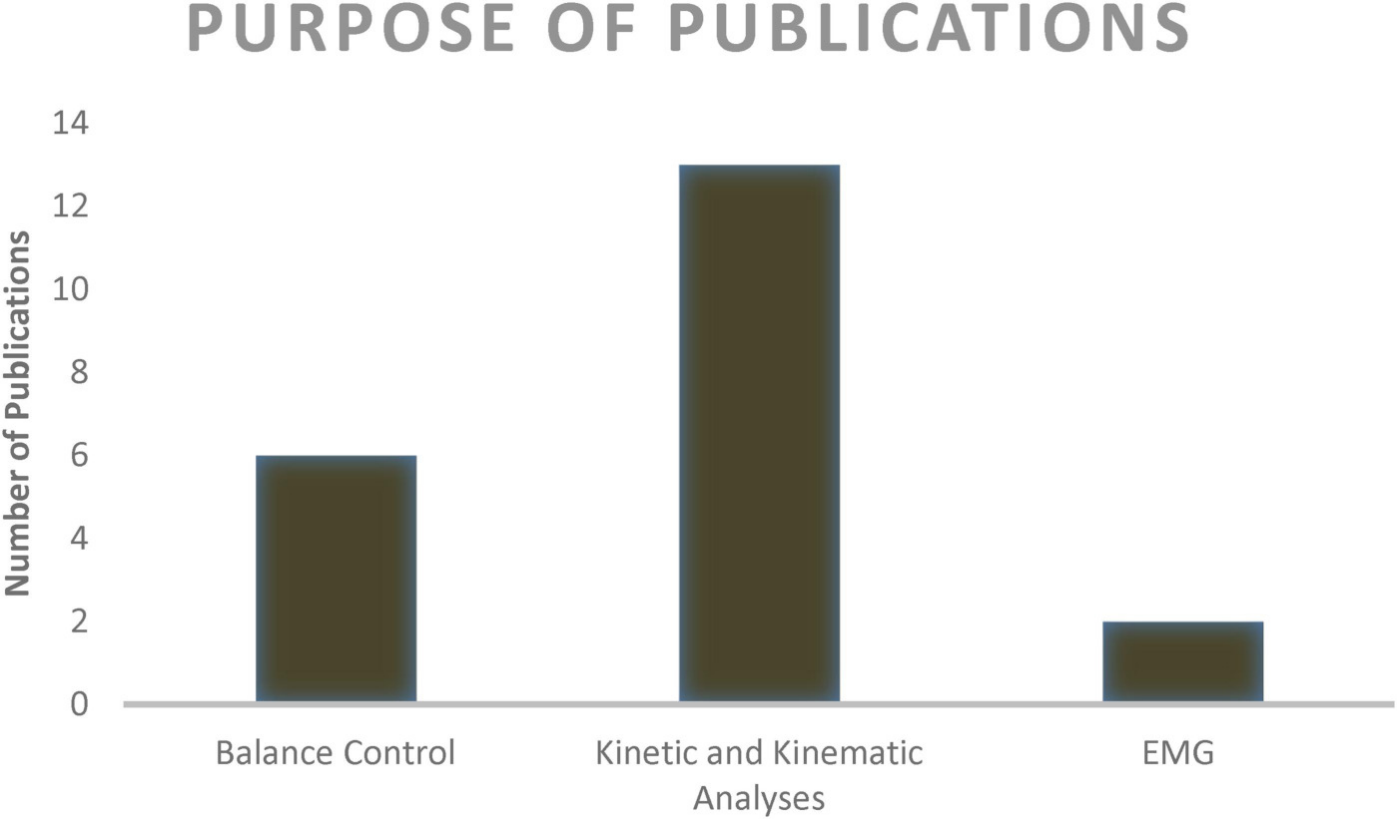
Overview of main objectives of all publications.

Figure 2 provides an overview of the objectives and topics examined in the studies included in this systematic review. Six studies focused on balance control, aiming to analyze the effects of head position and visual condition on balance control during the handstand (5, 6). Two studies aimed to determine characteristics of handstand balance (13, 14). One study compared stability indices between standing and handstand positions (21). Another study investigated control strategies used by gymnasts to maintain handstand balance (11). Among the remaining studies, 11 emphasized kinetic and kinematic analyses. One study proposed a biomechanical model of the press handstand, investigating shoulder joint torque requirements (22). Kerwin and Trewartha (12) analyzed strategies to maintain handstand balance in the anterior–posterior direction. Another study compared training loads between gymnasts of different participation levels (international vs. national) (16).

Veličković et al. (26) and (25) analyzed kinematic parameters in two investigations of the basket-to-handstand movement and the basket with a half-turn to the handstand on parallel bars. Their primary objective was to determine which kinematic parameters most strongly influenced successful basket-to-handstand performance. A more recent study by Velickovic and associates showed that successful gymnastics achieved enhanced performance velocities by optimizing optimal positioning, initiating early second-phase entry, and maximizing energy efficiency (28). Mizutori and colleagues demonstrated how skilled gymnasts used hip flexion, acute pike, and flexor moments to execute an effective press handstand (20). Two additional studies analyzed the kinematics of the basket-to-handstand movement and compared performance techniques between gymnasts with the highest and lowest performance levels (23, 24). Gautier et al. (17) investigated the influence of participation level among gymnasts on coordination patterns displayed during handstand performance. Kalichová et al. (2) analyzed the contribution of strength abilities to the quality of handstand performance, *n* = 1. Another study identified parameters related to successful motor control during the handstand (27).

Finally, Blenkinsop et al. (15) assessed the prevalent control strategies employed to retain balance during standing and handstand positions. In the electromyography studies, only two publications compared muscle activity among gymnasts. One study compared muscle activity patterns in adult and young gymnasts during the handstand. The other study analyzed changes in muscle activity during handstands performed on different apparatuses (18, 19).

#### Biomechanical analysis

Biomechanical analyses were conducted across all 21publications, which varied in their analytical approaches, chosen parameters, and variables. Among these, 42% analyzed the center of pressure (COP) (2, 5, 6, 13–15, 21, 27), 36% analyzed joint angles (6, 15, 22–26, 28), 21% reported on velocity (23–25, 27), 21% assessed joint torque contributions (11, 12, 15, 22), 15% investigated angular velocity (22, 25, 26), 10% analyzed ground reaction forces (GRFs) (14, 16) and moments (20), 10% reported on electromyography (EMG) findings (18, 19), and 5% analyzed vertical ground reaction forces (vGRFs) (14) (Table 2).

**Table 2:** Summary of research studies that examined the handstand.

Study	Purpose of the study	Characteristics	Biomechanical application	Body segments/muscles recorded	Main findings
(5)	Analyzed the effect of head position and visual condition on balance control of the handstand	Participants: three male and two female gymnasts Mean age: 21 ± 3 years; height: 165 ± 7 cm; and mass: 58 ± 9 kg	COP trajectories, AMTI force plates, and three-dimensional system of movement analysis	External malleolus, lateral femoral epicondyle, greater trochanter, elbow, gleno-humerus axis, styloid process of cubitus, 10th dorsal vertebra, and posterior and anterior parts of the summit of the cranium	Results illustrated that the neck reflexes played a significant role in maintaining balance within the handstand. It was reported that head standard and head dorsiflexion position facilitated balance within the handstand
(15)	Analyze prevalent control strategies attempted by gymnasts to retain balance in the handstand and standing postures during perturbed and unperturbed balance tasks	Participants: 12 experienced gymnasts Nine male gymnasts (age: 23.1 ± 3.6 years; weight: 69.9 ± 2.2 kg; height: 1.73 ± 0.05 m) Three female gymnasts (age: 20.5 ± 0.7 years; weight: 57.9 ± 1.9 kg; height: 1.64 ± 0.02 m)	Nine T20 Vicon cameras and force plates	Wrist, elbow, knee, and ankle	This study illustrated that distinct strategies are employed for unperturbed and perturbed balance. In the handstand, the predominant control strategy was the wrist strategy, and the standing control strategy involved the ankles. Wrist torque contributed to center of mass displacement and controlled the center of mass and velocity, with contributions from torques at the shoulder and hips Prevalent control strategy: Wrist, followed by elbow strategy, then a shoulder strategy in the handstand
(16)	Determine the effects of participation level (international vs. national), apparatus (beam and floor), and training phase (precompetition and competition) on estimates of training load in 25 female artistic gymnasts	Participants: 25 female artistic gymnasts (age: 9.5; SD = 1.6 years)	Video analysis and force platforms	Wrist and ankles	Results illustrated that ground reaction forces were lower within fundamental skills in national-level gymnasts compared to international gymnasts; international gymnasts experienced greater impacts than national-level gymnasts. Overall, it was emphasized that coaches must be aware of the increase in the magnitude of impacts and should follow a periodized training program to reduce the risk of injury among gymnastics
(6)	Increase our understanding of postural regulation by analyzing the handstand	Participants: 10 male gymnasts [age: 18–25 years; mean age: 22.1 years (s ¼ 3.0); mean height: 1.71 m (s ¼ 0.07); mean mass: 68.3 kg (s ¼ 9.5)]	Strain gauges, recorded variations in the center of pressure along the anterior–posterior (Y) and medial–lateral (X) axes	Left side of gymnasts on the joint centers of the wrist, elbow, shoulder, hip and knee	The main findings illustrated that balance was maintained mostly through the movement of shoulders and wrists; however, the hips hardly moved in better balance control handstands. It was also illustrated that when gymnasts failed to use the wrist strategy, flexion occurred. However, this decreased the center of gravity and resulted in imbalance
(17)	Investigate the effect of expertise on coordination patterns of the handstand	Participants: 16 male and female gymnasts	Vicon 512 system and the 3DSmax	Right side: middle finger of the right hand, wrist, elbow, shoulder, hip, and ankle	The main findings of this study illustrated that the more advanced the level of expertise demonstrated by the gymnasts, the better their suprapostural task performance. Gymnasts exhibited different balance coordination types in relation to their level of expertise. Generally, less experienced gymnasts led coordination patterns with their hips and demonstrated higher angular amplitude compared to expert gymnasts who controlled their handstand balance with their wrists and shoulders
(2)	Investigate the relationship between strength abilities and the handstand.	Participants: 19 Female gymnasts Age: 10–13	Dynamometric platform with four tenzometric force sensors	Anterior–posterior plane	Gymnasts with better strength abilities employed a three-segment balancing strategy; gymnasts with less strength abilities used the three-segment balancing strategy for short period of time and corrected their movement based on four-segment control strategy, illustrating that gymnasts with better strength abilities were able to employ the three-segment balancing strategy and hold balance for longer durations
(12)	Determine the contributions made by wrist, shoulder, and hip joint torques in maintaining the handstand	Participants: six male gymnasts (age: 15.7 ± 2.7 years; weight: 53.63 ± 10.04 kg; height: 1.62 ± 0.08 m)	2D capture system and force plates	Fingers, wrists, elbows, shoulders, hips, knees, ankles, toes, and the center of the head	This study illustrated that joint torques contributed to CM movement. Adequate handstands were characterized by significant contributions from the wrist and shoulder torques, with minimum influence from hip torque; when hip angles were greatly involved, balance degraded
(18)	Evaluate the relationship between muscle activity and intermuscle contribution patterns and postural control during a handstand. In addition, outcomes were compared between young and adult gymnasts	Participants: gymnasts (mean ± SD: 13.9 ± 0.7 and 23 ± 3 years)	Force platform and EMG	Trapezius descendens, latissimus dorsi, anterior deltoid, tricep brachii, bicep brachii, wrist extensors, wrist flexors, pectoralis major, multifidus, gluteus maximus, rectus abdominis, and rectus femoris	This study illustrated that adult and young gymnasts displayed different relationships between muscle activity and balance control The most activated muscles with the highest relative mean were the wrist flexors, which correlated with better postural control variables Young gymnasts had greater mean relative values in muscle activity in the triceps brachii, biceps brachii, and rectus femoris muscles compared to the adult group Young gymnasts displayed comparable muscle contributions during the handstand; however, they demonstrated different relationships between muscle activity and postural control regulations
(19)	Evaluate the difference in electromyography (EMG) findings between handstands performed on three apparatuses (floor, rings, and parallel bars) and the difference between young and well-trained adult gymnasts	Participants: 10 adult (age: 25 ± 3.94 years) and 15 young (13.9 ± 0.7 years) gymnasts participated in the study	EMG	Trapezius descendent, latissimus dorsi, lateral deltoid, anterior deltoid, triceps brachii, biceps brachii, wrist extensors, wrist flexors, pectoralis major (pars sternalis), multifidus, gluteus maximus, rectus abdominis, and rectus femoris	The main findings illustrated that gymnasts displayed different muscle activation patterns, which were dependent on hand support, altering wrist strategy with the activation of other muscles controlling balance
(20)	This study aimed to clarify the kinematics and joint moment profiles during straight arm press to handstand in different highly skilled male gymnasts	Participants: 59 male gymnastics [age: 16.4 ± 2.9 years, body height (BH): 155.1 ± 12.5 cm, body weight (BW): 50.6 ± 12.1 kg], including 48 junior elite national gymnasts in a national training camp	Force plate, video-camera (Basler AG)	Kinematic and kinetic outputs including COP and COM measurements, joint moments, body segment inertial properties, segment angles, and joint angles (wrist, shoulder, hip)	Highly skilled gymnasts differed from less skilled performers in the way they produced hip joint moments during the straight arm press to handstand. Skilled gymnasts showed a shift from hip extension to hip flexion as the body moved from the leg-horizontal position to the full handstand. Their successful technique was also characterized by adopting a more acute pike position at toe-off and generating hip flexor moments during the latter phase of the movement
(21)	The aim of this study was to compare and analyze of relationships between stability indices registered in two positions—standing and handstand—in athletes practicing gymnastics at various levels of advancement	Participants: 46 athletes	Force platforms	Displacement of the COP in the anterior–posterior direction Displacement of the COP in the medial–lateral direction	Results illustrated that the participation level of gymnastics differed in terms of stability in standing and the handstand. More experienced gymnasts demonstrated better control over displacement of the center of pressure
(22)	Biomechanical model of the press handstand was developed to evaluate and predict the shoulder joint torque requirements and the motion of a gymnast's center of mass (CM) from an initial to a final (handstand) position	Participants: 5 gymnasts	3D motion capture system	Wrist, shoulder, hip, and ankle joints	The main findings illustrated that fewer fluctuations in body segment angular velocity required smoother and larger shoulder joint torques. Hip joint reductions by 5° or 10° do not reduce the shoulder joint torque requirements. Modification in the wrist joint angle is necessary to maintain center of mass above the center of the wrist joint, in which it lags the joint during the first phase of the movement and moves in front of the joint at the later stages of the press to handstand; results highlighted that limiting factors include poor motor coordination as opposed to strength abilities
(13)	The aim of the paper was to analyze the process of maintaining body balance during the handstand and one-leg toe stance	Participants: 10 athletes, including five girls and five boys	Kistler force platforms	The displacement of COP was recorded in two planes: sagittal (anterior–posterior) and frontal (medial–lateral)	It was illustrated that the maintenance of balance is executed mainly in the anterior–posterior direction; COP was found to be more important for body stability
(14)	This study aimed to determine the characteristic features of handstand posture control associated with a high level of ability among male gymnasts	Participants: eight acrobatic gymnasts	AccuSway (AMTI) force platform	Right and left hands	The main findings within this study illustrated that from a biomechanical analysis, less skillful gymnasts exerted pressure on the surface by the hands in the medial–lateral direction. Experienced gymnasts were able to control the body more fluently and recorded less variation of body sway
(23)	Analyze the new outer grip and examine whether it achieves higher body angle and center of gravity than the inner grip techniques at the moment of bar regrasp	Participants: 25 American gymnasts and 28 Japanese gymnasts	2D motion capture system	Shoulder, hip, and elbow joints	This study highlighted that the new outer grip technique had a technical advantage for pursuing virtuosity points and new maneuvers
(24)	Compare differences between kinematics across gymnasts from two different groups within phases of basket-to-handstand movement	Participants: 26 gymnasts from 25 American and 28 Japanese gymnasts	A 16-mm LoCam II DC camera	Horizontal and vertical coordinates of 21 points and a 14-segment model of the body	The main findings from this study illustrated that, from biomechanical analysis, the most successful performance was achieved when the arm pull is sustained with maximum effort until the body center mass and the trunk are above the bars to release with high body center mass, high vertical velocity, backward horizontal velocity, and forward angular momentum
(26)	Conduct a kinematic analysis of the basket-to-handstand movement	One experienced gymnast (age: 26 years; weight: 63 kg; height: 1.65 m)	APAS 3D video system	Tip of the right foot, center of the right shoulder joint, center of the right hip joint, and top of the head	This study defined a kinematic model for the basket-to-handstand movement, in which it was reported that it requires four phases: (1) upswing from a handstand, (2) downswing to upswing, (3) forward swing to upswing, and (4) downswing to the handstand
(25)	Conduct a kinematic analysis of the basket with half-turn to the handstand on parallel bars	One experienced gymnast (age: 26 years; weight: 63 kg; height: 1.65 m)	3D video system	Tip of right foot, right shoulder joint, hip joint, and top of the head	This study defined a kinematic model for the basket with half-turn to the handstand on parallel bars, in which four subphases were modeled
(27)	The purpose of this study was to identify parameters that are linked with successful motor control within the handstand	Participants: Competitive female gymnasts (age: 10 ± 1 years; height: 1.37 ± 0.09 m; weight: 31.5 ± 2.9 kg)	3D motion analysis system and Kistler force plates	Whole body center of mass	Main findings from this study illustrated that characteristics of center of mass (COM) and center of pressure (COP) for experienced and less experienced gymnasts were found to be reliant in the anterior–posterior and medial–lateral directions
(11)	Investigated the control strategy employed by gymnasts in maintaining handstandbalance	Participants: four male gymnasts (age: 16.5 ± 2.9 years; weight = 52.4 ± 10.0 kg; height = 1.61 ± 0.07 m)	Force plate (Kistler 9281-B12) and video camera recorder systems	Posterior surface of the body	Main findings from this study illustrated that the wrist torque had a strong relationship with the mass center displacement and velocity within the handstand. The directions of the shoulder and hip torques were the same as those of the wrist torque, which is in accordance with the use of the wrist strategy
(28)	This study aimed to examine the differences in the kinetic energy of the body's center of mass between successful and unsuccessful attempts at transitioning from a basket to a handstand on parallel bars	Participant: one elite gymnast (age: 26 years; body height: 165 cm; body weight: 63 kg)	Parallel bars, 2D Casio digital camera EX-F1, and Ariel Performance 3D analysis system	Total kinetic energy (KE_total_), kinetic energy of translational motion (KE_trans_), and kinetic energy of rotational motion (KE_rot_)	Successful gymnastics attempts involve better biomechanics and efficient energy use. Entering the second phase earlier, with higher feet and hips and a smaller shoulder angle, improves kinetic chain efficiency. This leads to greater peripheral and angular velocities, which are vital for executing elements effectively

COP, center of pressure; COM, center of mass; EMG, electromyography.

#### Electromyography

**Figure 3:**
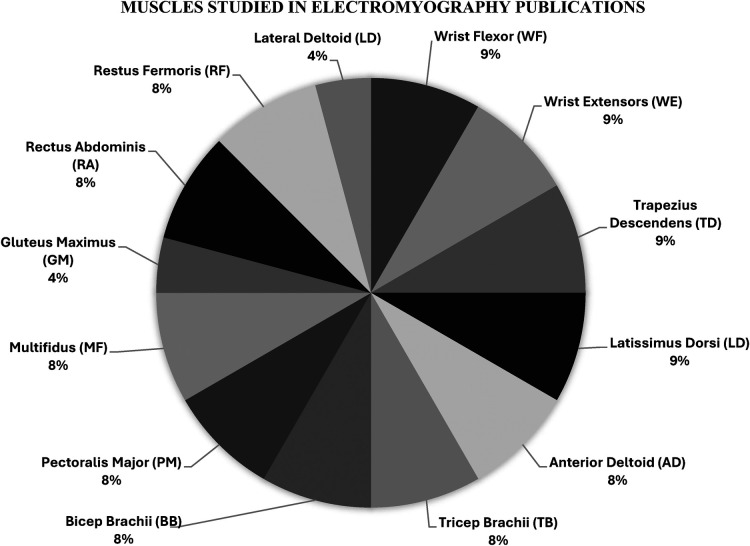
Percentage of muscles investigated in electromyography studies.

 Figure 3 illustrates the frequency of muscles investigated across the studies. Of the 19 studies, only two included electromyography analysis. These two studies investigated the following muscles: wrist flexors (WFs), wrist extensors (WEs), trapezius descendens (TD), latissimus dorsi (LD), anterior deltoid (AD), tricep brachii (TB), bicep brachii (BB), pectoralis major (PM), multifidus (MF), rectus abdominis (RA), and rectus femoris. One study investigated the lateral deltoid and gluteus maximus (GM) (18, 19).

## Discussion

### Balance control strategies via biomechanical analysis

Biomechanical analyses provided a pathway for investigators to thoroughly analyze balance control strategies during handstand performance, which accounted for 52% of all publications. The strategies examined included COP movement analysis with an integrated assessment of head position, visual condition, joint angles, GRFs, and joint torques (4–6, 11–15, 21). In 21% of the studies with similar objectives and results, the papers highlighted the role of the “wrist strategy” employed by gymnasts to maintain balance during the handstand (6, 11–13, 15). Yeadon and Trewartha (11) and Gautier et al. (6) reported undifferentiated results. Both studies utilized two-dimensional motion capture systems synchronized with force plates. They concluded that the greatest activation occurred in the wrists, with the wrist flexors and extensors correcting the balance control in the sagittal plane. This was done synergistically through contributions from the shoulder and hip joints, which help maintain balance. Rotations at the shoulder and hips occurred in the same direction as those at the wrists. If gymnasts failed to use the “wrist strategy,” it resulted in flexion of the elbow. Flexion of the elbow in the handstand results in extreme imbalance and displacement of the center of gravity (6).

Moreover, similar studies found that wrist action correlated with better balance control. Biomechanical analyses revealed that the COP shifted toward the wrists or fingers in the sagittal plane, increasing pressure under the wrist joints as the center of gravity shifted toward the wrist, an effect correlating with better balance control and improved technique. Overall, wrist and shoulder torques improved balance, whereas hip torques had a minimal influence on balance (12, 13). In a related study investigating balance control strategies in perturbed and unperturbed standing conditions, the most prevalent strategies were the “wrist strategy” and the “ankle strategy.” In contrast to previous publications, the “wrist strategy” was employed for more than 75% of the time during the handstand. This can be categorized as a single-segment inverted pendulum control strategy, as opposed to other methodologies that emphasize contributions of shoulder and hip torques, which can be classified as a mixed strategy. This single-segment inverted pendulum control strategy describes a multisegment system primarily controlled by torque at the most inferior joint, with torques at superior joints acting in the same direction to maintain a fixed-body configuration (15).

In brief, Yeadon and Trewartha (11) investigated wrist-dominant balance control, Wyatt et al. (27) examined bidirectional pressure control, and Pryhoda et al. (29) analyzed task-specific joint variability. Collectively, these three studies advance our understanding of the neuromechanical control strategies underpinning human balance in both upright and inverted postures. In particular, Yeadon and Trewartha (11) identified a wrist-dominant control strategy in handstands, in which wrist torque responds to deviations in body position and velocity, supported by coordinated shoulder and hip actions to maintain a fixed-body configuration. Wyatt et al. (27) extended this by demonstrating that skilled handstand balance involves bidirectional causal interactions between the center of pressure (CoP) and the center of mass (CoM), with more proficient gymnasts exhibiting stronger CoP-to-CoM influence, particularly in the anterior–posterior direction. This suggests that effective handstand control is not merely reactive but relies on anticipatory adjustments. Complementing these findings, Pryhoda et al. (29) showed that joint motion variability is both task-specific and governed by general motor control principles. In upright stance, lower-limb joints (ankle, knee, and hip) dominate variability, whereas in handstands, upper-limb joints (wrist, elbow, and shoulder) take precedence. Together, these studies underscore that balance control is a dynamic, joint-specific process shaped by task demands and skill level. These results highlight the importance of adaptive variability and coordinated joint action in achieving postural stability, offering valuable insights for training, rehabilitation, and development of bio-inspired control systems.

### Effect of head positions and visual conditions

Approximately 10% of the studies incorporated visual conditions and head positions for balance control during the handstand. One study investigated the role of peripheral vision and central vision in balance control. Using a stabilometric platform equipped with measurement sensors, researchers analyzed COP variations in the frontal (X) and sagittal (Y) planes, as well as angular relationships between body segments. It was concluded that in eye-closed trials, balance deteriorated and COP increased (6). In another unique study, the effect of visual condition and head position was analyzed on five experienced gymnasts who each performed three handstands for maximum duration on AMTI force plates equipped with strain gauges synchronized with three-dimensional motion capture systems. The systems analyzed the COP and kinematics of different body segments. The gymnasts performed handstands under four different head positions (Hd, Hv, Ha, and Hs), with eyes open and eyes closed. The study concluded that the tonic neck reflex plays a crucial role in balance control during handstands. Although no significant kinematic differences were observed, it was concluded that both the standard head position and head dorsiflexion significantly influenced balance control in both eyes-open and eyes-closed trials. Gymnasts struggled to maintain balance when their neck was in flexion, likely due to changes in head orientation and vestibular apparatus (5).

#### Characteristics

Previous literature focused on the mechanics of the handstand. However, more recent studies have increasingly considered specific characteristics, i.e., age, gender, participation level, and strength abilities, in relation to the handstand technique. One unique study concluded that gymnasts with better strength values in strength performance tests exhibited superior balance control, maintaining the handstand position for a longer duration, and demonstrated better technique compared to gymnasts with limited strength abilities. Interestingly, this study found that gymnasts with greater strength utilized specific balance control strategies, such as a three-segment strategy, compared to those with lower strength abilities, who were only able to maintain the three-segment control strategy for a shorter duration. When fatigue set in and they could no longer sustain the position, they shifted to a four-segment control strategy (employing the elbows), which resulted in imbalance (2). Furthermore, researchers have emphasized the importance of strength in balance control, noting that joint movements are governed by a chain of muscle activation patterns (30). This activity requires significant upper-body strength to support body weight, particularly the shoulder girdle (31).

Studies have also examined participation levels and their impact on handstand performance outcomes (14, 17, 21). In two previous studies, where less experienced gymnasts were compared to those with greater experience, the latter displayed better values for COP in the hands, the mean amplitude of COP, and COP in the medial–lateral direction (14, 21). Sobera et al. (14) further concluded that junior gymnasts displayed no relationship between stability indicators and showed higher values of COP displacement. This was likely due to angular displacements in the joints. Typically, a fixed-body configuration is improved by reducing angular displacement in the hip, shoulder, and elbow joints, while ensuring precise activation of the radiocarpal joints and specific muscles associated with the talar joint and toes. Overall, junior gymnasts displayed a lack of strength in the specific muscles responsible for maintaining stability during handstand performance (14). A further study compared coordination patterns during handstand performance across different expertise levels. Overall, it was observed that higher-level gymnasts demonstrated better handstand performance. Gautier et al. (17) concluded that specific coordination patterns influenced balance control during the handstand. Non-elite gymnasts coordinated their handstand with their hips, displaying higher angular amplitudes compared to elite gymnasts, who controlled their handstand using their wrists and shoulders with minimal angular movement of the hips. Over time, elite gymnasts demonstrated adapted coordination patterns, underscoring the effect of training and experience on performance (17).

Researchers have also compared the relationship between postural control and muscle activity during handstand performance in young and adult gymnasts. All gymnasts performed handstands on force platforms, while electromyography recorded muscle activity of the lower and upper limbs. This study concluded that adult gymnasts demonstrated better postural control, with the wrist flexors showing the highest relative mean values among all muscle group studies. In contrast, younger gymnasts displayed different muscle activity patterns, demonstrating greater relative mean values for the triceps, biceps, and rectus femoris muscles. The study concluded that these differences may be the result of training performance and preparation rather than age (19, 32). For young gymnasts, the overall aim during the handstand is to minimize movement across body segments by controlling changes in the limb and torso. This desirable outcome can normally be achieved within 2 years of frequent training (13).

#### Limitations

This systematic review has a few limitations. Only two publications analyzed electromyography data. Future research should pay more focus on electromyography using an integrated 3D motion capture system. This can provide critical information on COP movement analysis corresponding with specific muscle activation patterns. There was also a scarcity of studies investigating strength abilities and their influence on balance control—only one study addressed this aspect. Further research is required to develop appropriate and specific strength tests for identifying the strength parameters required for gymnastic performance. Finally, because the included studies analyzed different parameters and pursued varying research objectives, it was challenging to compare their significant differences.

## Conclusion

This systematic review outlines the biomechanical analysis of the handstand. Balance control is a dominant feature, and it is predominantly assessed based on shifts in the COP. To maintain balance, gymnasts most often employ a “wrist strategy” to retain a fixed-body configuration that controls body sway by maneuvering movement in the wrist joint while minimizing movement in other body segments. If the “wrist strategy” fails, then the wrists, shoulder, hips, and elbows are employed, which can be characterized as a mixed control strategy. The wrists are crucial for achieving maximal balance during handstand performance. The COP shifts toward the wrists or fingers in the sagittal plane, increasing pressure under the wrist joints as the center of gravity moves toward the wrist. Moreover, different systems become activated, providing the gymnasts with signals and information from the visual, proprioceptive, and vestibular cues.

Visual conditions and head positions influence balance during handstand performance. When the eyes are closed, stability decreases, and neck flexion further contributes to loss of balance during handstand performance. Strength also plays a key role in determining the quality of the handstand. Gymnasts with greater strength display better postural control compared to those with minimal strength. Other factors influencing handstand performance are participation level, age, and experience. In addition, adult gymnasts exhibited better balance control during handstand performance across all reviewed publications.

## Data Availability

The original contributions presented in the study are included in the article/Supplementary Material; further inquiries can be directed to the corresponding author.

## References

[1] Rohleder J Vogt T. Performance control in handstands: challenging entrenched coaching strategies for young gymnasts. Int J Perform Anal Sport. (2018) 18:17–31. 10.1080/24748668.2018.1440459

[2] Kalichová M Hedbávný P Bago G. Influence of strength abilities on quality of the handstand. Int J Sport Health Sci. (2013) 7:602–8.

[3] Slobounov SM Newell KM. Postural dynamics in upright and inverted stances. J Appl Biomech. (1996) 12:185–96. 10.1123/jab.12.2.185

[4] Hedbávný P Sklenaříková J Hupka D Kalichová M. Balancing in handstand on the floor (Ohranjanje Ravnotežja v Stoji Na Rokah Na Parterju). Sci Gymnast J. (2013) 5:69–79.

[5] Asseman F Gahéry Y. Effect of head position and visual condition on balance control in inverted stance. Neurosci Lett. (2005) 375:134–7. 10.1016/j.neulet.2004.10.085 15670656

[6] Gautier G Thouvarecq R Chollet D. Visual and postural control of an arbitrary posture: the handstand. J Sports Sci. (2007) 25:1271–8. 10.1080/02640410601049144 17654239

[7] Zalewski CK. Aging of the human vestibular system. Semin Hear. (2015) 36:175–96. 10.1055/s-0035-1555120 27516717 PMC4906308

[8] Jerosch J Prymka M. Proprioception and joint stability. Knee Surg Sports Traumatol Arthrosc. (1996) 4:171–9. 10.1007/BF01577413 8961235

[9] Vuillerme N Teasdale N Nougier V. The effect of expertise in gymnastics on proprioceptive sensory integration in human subjects. Neurosci Lett. (2001) 311:73–6. 10.1016/S0304-3940(01)02147-4 11567781

[10] Uzunov V. The handstand: a four stage training model. Gym Coach. (2008) 2:52–9.

[11] Yeadon MR Trewartha G. Control strategy for a hand balance. Motor Control. (2003) 7(4):411–30. 10.1123/mcj.7.4.421 14999137

[12] Kerwin DG Trewartha G. Strategies for maintaining a handstand in the anterior-posterior direction. Med Sci Sports Exerc. (2001) 33:1182–8. 10.1097/00005768-200107000-00016 11445766

[13] Sobera M Siedlecka B Piestrak P Sojka-Krawiec K Graczykowska B. Maintaining body balance in extreme positions. Biol Sport. (2007) 24:81–8.

[14] Sobera M Serafin R Rutkowska-Kucharska A. Stabilometric profile of handstand technique in male gymnasts. Acta Bioeng Biomech. (2019) 21:63–71. 31197281

[15] Blenkinsop GM Pain MTG Hiley MJ. Balance control strategies during perturbed and unperturbed balance in standing and handstand. R Soc Open Sci. (2017) 4(7):161018. Published July 26, 2017. 10.1098/rsos.161018 28791131 PMC5541526

[16] Burt LA Naughton GA Higham DG Landeo R. Training load in pre-pubertal female artistic gymnastics (Trenažne Obremenitve Telovadk V Predpubertetnem Obdobju). Sci Gymnast J. (2010) 2:5–14. 10.52165/sgj.2.3.5-14

[17] Gautier G Marin L Leroy D Thouvarecq R. Dynamics of expertise level: coordination in handstand. Hum Mov Sci. (2009) 28:129–40. 10.1016/j.humov.2008.05.003 18986720

[18] Kochanowicz A Niespodziński B Marina M Mieszkowski J Biskup L Kochanowicz K. Relationship between postural control and muscle activity during a handstand in young and adult gymnasts. Hum Mov Sci. (2018) 58:195–204. 10.1016/j.humov.2018.02.007 29471194

[19] Kochanowicz A Niespodziński B Mieszkowski J Marina M Kochanowicz K Zasada M. Changes in the muscle activity of gymnasts during a handstand on various apparatus. J Strength Cond Res. (2019) 33:1609–18. 10.1519/JSC.0000000000002124 28700510

[20] Mizutori H Kashiwagi Y Hakamada N Tachibana Y Funato K. Kinematics and joints moments profile during straight arm press to handstand in male gymnasts. PLoS One. (2021) 16(7):e0253951. 10.1371/journal.pone.0253951 34260617 PMC8279359

[21] Omorczyk J Bujas P Puszczałowska-Lizis E Biskup L. Balance in handstand and postural stability in standing position in athletes practicing gymnastics. Acta Bioeng Biomech. (2018) 20:139–47. 30220715

[22] Prassas SG. Biomechanical model of the press handstand in gymnastics. Int J Sport Biomech. (1988) 4:326–41. 10.1123/ijsb.4.4.326

[23] Takei Y Dunn JH Nohara H Kamimura M. New outer grip technique used by elite gymnasts in performing the felge to handstand mount. J Appl Biomech. (1995) 11:188–204. 10.1123/jab.11.2.188

[24] Takei Y Dunn JH. A comparison of techniques used by elite gymnasts in performing the basket-to-handstand mount. J Sports Sci. (1996) 14:269–79. 10.1080/02640419608727710 8809718

[25] Veličković S Kolar E Petković D. The kinematic model of the basket with ½ turn to handstand on the parallel bars (Kinematički Model Kovrtljaja Nazad Iz Stava u Uporu Do Stava u Uporu Sa ½ Okreta Na Razboju Bočno). Facta Univ. (2006) 4:137–52.

[26] Veličković S Kolar E Kugovnik O Petković D Petković E Bubanj S The kinematic model of the basket to handstand on the parallel bars (Kinematički Model Kovrtljaja Nazad Iz Stava u Uporu Do Stava u Uporu Na Paralelnom Razboju). Facta Universitatis. (2011) 9:55–68.

[27] Wyatt HE Vicinanza D Newell KM Irwin G Williams GKR. Bidirectional causal control in the dynamics of handstand balance. Sci Rep. (2021) 11(1):405. 10.1038/s41598-020-79730-z 33432011 PMC7801474

[28] Veličković S Đorđević D Veličković P Možnik M Kolar E Stoica CE An analysis of the kinetic energy in the basket to handstand on parallel bars: a case study of an elite gymnast. Life (Basel, Switzerland). (2025) 15(2):172. 40003581 10.3390/life15020172 PMC11856254

[29] Pryhoda M Newell KM Wilson C Irwin G. Task specific and general patterns of joint motion variability in upright- and hand-standing postures. Entropy. (2022) 24(7):909. 10.3390/e24070909 35885134 PMC9323647

[30] Hayes KC. Biomechanics of postural control. Exerc Sport Sci Rev. (1982) 10:363–91. 10.1249/00003677-198201000-00011 6749518

[31] Beličič B Samardžija Pavletič M. Handstand on force plate in artistic gymnastics. 2nd International Scientific Congress Slovenian Gymnastics Federation (2015). p. 127.

[32] Dotan R Mitchell CJ Cohen R Gabriel D Klentrou P Falk B. Explosive sport training and torque kinetics in children. Appl Physiol Nutr Meta. (2013) 38:740–5. 10.1139/apnm-2012-0330 23980732

[33] Law M Stewart D Letts L Pollock N Bosch J Westmorland M. Guidelines for Critical Review of Qualitative Studies. Hamilton, ON: McMaster University Occupational Therapy Evidence-Based Practice Research Group (1998).

